# The Metabolic Switch of Physical Activity in Non-Obese Insulin Resistant Individuals

**DOI:** 10.3390/ijms24097816

**Published:** 2023-04-25

**Authors:** Shamma Almuraikhy, Najeha Anwardeen, Asmma Doudin, Maha Sellami, Alexander Domling, Abdelali Agouni, Asmaa A. Al Thani, Mohamed A. Elrayess

**Affiliations:** 1 Biomedical Research Center, Qatar University, Doha P.O. Box 2713, Qatar; 2 Groningen Research Institute of Pharmacy, Drug Design, Groningen University, 9713 AV Groningen, The Netherlands; 3 Physical Education Department (PE), College of Education, Qatar University, Doha P.O. Box 2713, Qatar; 4 College of Pharmacy, QU Health, Qatar University, Doha P.O. Box 2713, Qatar; 5 Department of Biomedical Sciences, College of Health Science, QU Health, Qatar University, Doha P.O. Box 2713, Qatar

**Keywords:** physical activity, insulin sensitive, insulin resistant, non-obese

## Abstract

Healthy non-obese insulin resistant (IR) individuals are at higher risk of metabolic syndrome. The metabolic signature of the increased risk was previously determined. Physical activity can lower the risk of insulin resistance, but the underlying metabolic pathways remain to be determined. In this study, the common and unique metabolic signatures of insulin sensitive (IS) and IR individuals in active and sedentary individuals were determined. Data from 305 young, aged 20–30, non-obese participants from Qatar biobank, were analyzed. The homeostatic model assessment of insulin resistance (HOMA-IR) and physical activity questionnaires were utilized to classify participants into four groups: Active Insulin Sensitive (ISA, n = 30), Active Insulin Resistant (IRA, n = 20), Sedentary Insulin Sensitive (ISS, n = 21) and Sedentary Insulin Resistant (SIR, n = 23). Differences in the levels of 1000 metabolites between insulin sensitive and insulin resistant individuals in both active and sedentary groups were compared using orthogonal partial least square discriminate analysis (OPLS-DA) and linear models. The study indicated significant differences in fatty acids between individuals with insulin sensitivity and insulin resistance who engaged in physical activity, including monohydroxy, dicarboxylate, medium and long chain, mono and polyunsaturated fatty acids. On the other hand, the sedentary group showed changes in carbohydrates, specifically glucose and pyruvate. Both groups exhibited alterations in 1-carboxyethylphenylalanine. The study revealed different metabolic signature in insulin resistant individuals depending on their physical activity status. Specifically, the active group showed changes in lipid metabolism, while the sedentary group showed alterations in glucose metabolism. These metabolic discrepancies demonstrate the beneficial impact of moderate physical activity on high risk insulin resistant healthy non-obese individuals by flipping their metabolic pathways from glucose based to fat based, ultimately leading to improved health outcomes. The results of this study carry significant implications for the prevention and treatment of metabolic syndrome in non-obese individuals.

## 1. Introduction

Metabolic syndrome is a group of conditions caused by an unhealthy diet and lack of physical activity, which increases the risk of various diseases, including type 2 diabetes (T2D), heart disease, and stroke. [1]. The apparently healthy non-obese insulin resistant (IR) individuals are at higher risk of developing metabolic syndrome [2,3,4]. Studies have revealed that 40% of lean/overweight young females in Qatar are IR, which is a higher percentage compared to 25% of overweight females in other ethnic groups [5,6,7]. IR causes changes in how the body uses energy, leading to increased levels of free fatty acids, glucose and amino acids [8,9,10]. This results in higher insulin secretion to maintain normal glycaemia.

The metabolic signature associated with the increased risk was previously determined. A recent study has identified several potential metabolic biomarkers of insulin resistance, including amino acids (Asn, Gln, and His), methionine sulfoxide, 2-methyl-3-hydroxy-5-formylpyridine-4-carboxylate, serotonin, L-2-amino-3-oxobutanoic acid, and 4,6-dihydroxyquinoline [11]. Another study reported 113 out of 229 metabolic measures linked to T2D in a meta-analysis of four cohorts, with strong biomarkers being branched-chain and aromatic amino acids, triacylglycerol within VLDL particles, linoleic n-6 fatty acid, and non-esterified cholesterol in large HDL particles. A multi-metabolite score of phenylalanine, non-esterified cholesterol in large HDL, and the ratio of cholesteryl ester to total lipid in large VLDL was associated with future diabetes risk (odds ratio of 10.1) in one of the cohorts (mean age 31 years) and was more strongly associated with deterioration in post-load glucose and insulin resistance than with future fasting hyperglycaemia [12]. 

The metabolic switch refers to the body’s transition in energy utilization from glucose obtained through glycogenolysis to fatty acids and fatty acid-derived ketones. There is a growing body of evidence indicating that ketones are the preferred fuel for both the brain and body during fasting and prolonged exercise. This switch involves moving away from lipid synthesis and fat storage towards mobilization of fat through free fatty acids (FFAs) and fatty-acid derived ketones. As a result, when the metabolic switch occurs, the primary source of energy for the body shifts from glucose to FFAs obtained from adipose tissue lipolysis and ketones, preserving muscle mass [13]. Periodic flipping of metabolic switching not only provides ketone bodies as an energy source during fasting, but it also regulates the expression of various proteins and molecules that impact health and aging [14].

Physical inactivity is a significant risk factor for various metabolic conditions such as insulin resistance, T2D and cardiovascular disease [15,16], but the specific metabolic pathways through which physical activity protects against these conditions are not fully understood [4]. Regular physical exercise improves blood sugar regulation and insulin sensitivity [17] and a healthy lifestyle can improve the lives of diabetic patients [16,18,19]. A 5-year controlled diet and exercise plan can lower the risk of T2DM in non-obese individuals with glucose intolerance [4]. Recent research in both rats [20,21] and humans [22] has shown that sensitivity of skeletal muscle to insulin increases after both acute exercise and physical training. Other studies have shown that regular exercise can improve glucose tolerance, insulin sensitivity, lipid parameters, blood pressure, and fibrinolytic activity in IR individuals [22,23,24]. The American College of Sports Medicine and American Heart Association recommend 150 min of moderate activity (30 min, 5 days/week) or 60 min of vigorous physical activity per week for adults, with a focus on regular walking to improve sugar metabolism, cardiorespiratory fitness, and overall quality of life [25] 

Exercise demands metabolic flexibility in order to match fuel availability with the metabolic process to meet the significant increases in energy needs. The ability to switch between glucose and fatty acid catabolism during short-term exercise in healthy individuals primarily depends on the intensity and duration of the exercise. Higher intensity exercise increasingly relies on glucose oxidation through oxidative phosphorylation, as well as anaerobic glycolysis at even higher intensities. This takes place regardless of insulin levels, which are typically low during exercise. As exercise intensity increases, fatty acid oxidation contributes less to overall energy supply. However, as exercise duration becomes longer, fatty acids play a larger role to overall energy supply [26]. Exercise physiology research has made progress in identifying the mechanisms responsible for altered fuel metabolism in obesity and diabetes, as well as exploring ways to enhance metabolic flexibility in skeletal muscle and adipose tissue. The ultimate goal is to prevent and treat metabolic disorders [26].

There is limited research on the metabolic pathways underlying the protective effects of moderate physical activity in healthy, non-obese individuals and how they differ between IS and IR counterparts. Understanding the metabolic signature of these groups could aid in understanding and preventing the development of metabolic diseases, like T2D, through targeted diet and exercise [27,28,29]. Metabolomics offers a quantitative measurement of metabolic profiles associated with exercise in moderately active non-obese participants to identify biomarkers and detect changes in response to various physiological states between IS and IR subjects [30,31]. This study aims to compare the metabolic profiles of IS and IR individuals in active and sedentary non-obese apparently healthy individuals to further explore the effect of moderate physical activity on high risk IR individuals compared to their sedentary counterparts.

## 2. Results

### 2.1. Comparing the Effect of Physical Activity on Clinical Traits between IS and IR Subjects

Table 1 reveals that the active group had significantly higher levels of C Peptide and triglycerides in the IRA compared to the ISA (*p* ≤ 0.05). Conversely, the sedentary group had higher levels of C Peptide, creatinine, uric acid, creatine kinase, creatine kinase 1, total protein, alkaline phosphatase, alanine transaminase, aspartate aminotransferase, and GGT 2 in the IRS compared to the ISS.

### 2.2. Multivariate Analysis of Metabolites Differentiating IS and IR in Sedentary and Active Non-Obese Subjects

**Figure 1:**
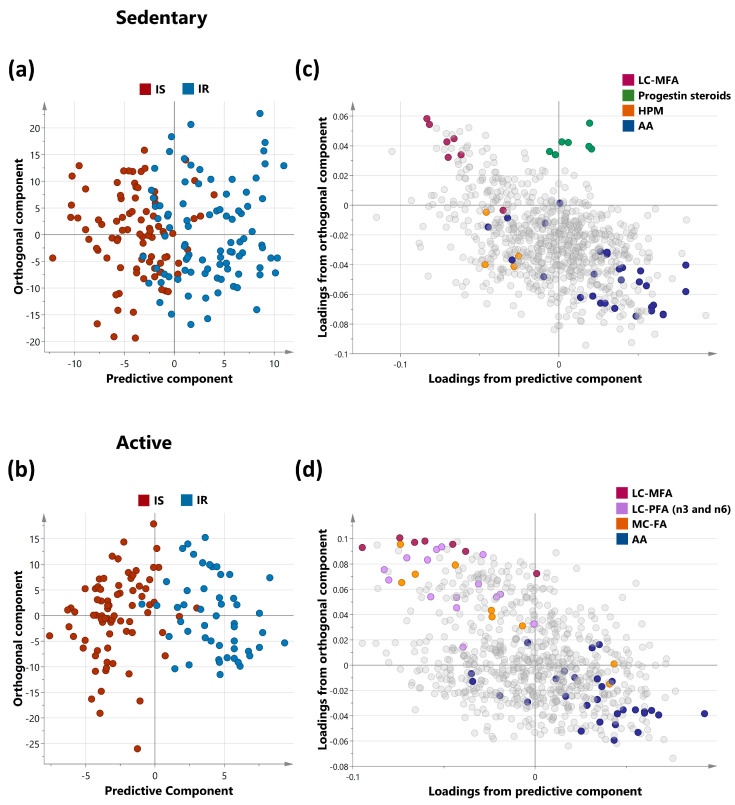
OPLS-DA of metabolites differentiating IS and IR in sedentary (**a**) and active (**b**) non-obese individuals. OPLS-DA (Orthogonal Projections to Latent Structures Discriminant Analysis) of insulin-sensitive and insulin-resistant individuals. The analysis was performed on sedentary and active cohorts separately as shown in score plots (**a**,**b**) for the two groups respectively. Model (**a**) (sedentary) identified one predictive and one orthogonal component while Model (**b**) (active) identified 1 predictive and 2 orthogonal components. (**c**,**d**) represent the corresponding loadings plots for sedentary and active individuals respectively. Enriched sub-pathways are coloured in both OPLS-DA models, including LCMFA—Long chain monosaturated fatty acids; LCPFA—Long chain polyunsaturated fatty acid, HPM—Haemoglobin and Porphyrin metabolism, MCFA—Medium chain fatty acid, AA—Amino acid (Leucine, Isoleucine and Valine metabolism).

The metabolic signatures of 305 subjects were analyzed using non-targeted metabolomics. OPLS-DA was utilized to identify the best distinguishing components between IS and IR sedentary and active groups, as shown in Figure 1a,b. The sedentary group had one predictive and one orthogonal component, with the discriminatory component accounting for 53.9% of the variance between ISS and IRS (R2X = 0.129; R2Y = 0.539; Q2 = 0.241). The active group had one predictive and two orthogonal components, with the discriminatory component accounting for 74.5% of the variance between ISA and IRA (R2X = 0.148; R2Y = 0.745; Q2 = 0.188). The loading plots showed which sets of metabolites best differentiated between sedentary and active individuals, including Long chain monosaturated fatty acids (LCMFA), Long chain polyunsaturated fatty acid (LCPFA), Haemoglobin and Porphyrin metabolism (HPM), Medium chain fatty acid (MCFA), and Amino acid (AA) metabolism (Leucine, Isoleucine and Valine). These are shown in the loading plots for sedentary and active individuals in Figure 1c,d. Mulitvariate analysis of all four groups was also performed. Corresponding scores plot and list of metabolites with VIP > 1.5 are summerized in Supplementary Figure S1 and Table S1, respectively.

### 2.3. Univariate Analysis of Metabolites Differentiating Sedentary Insulin Sensitive and Insulin Resistant Individuals

**Figure 2:**
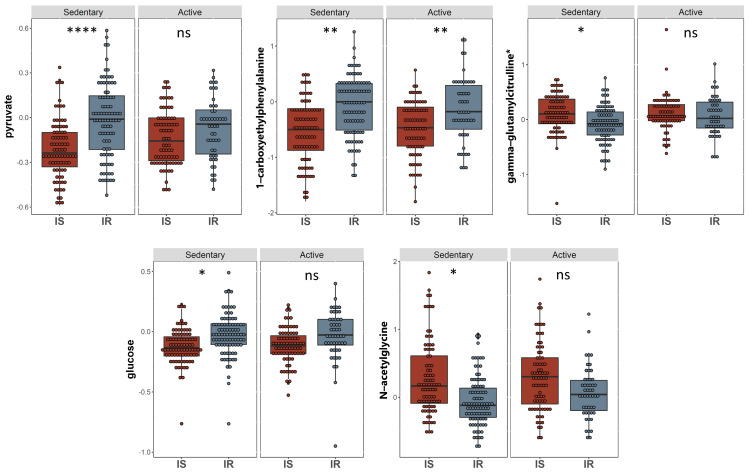
Boxplots of the top hits from the linear regression and functional enrichment analysis between insulin sensitive and insulin resistant in sedentary individuals. (I) Boxplots representing the metabolites with significantly different levels (FDR ≤ 0.05), ****/**/* signifies <0.0001/<0.01/<0.05 after adjusting for multiple correction using FDR method. Y-axis indicates level of metabolites in log_e_ scale.

**Figure 3:**
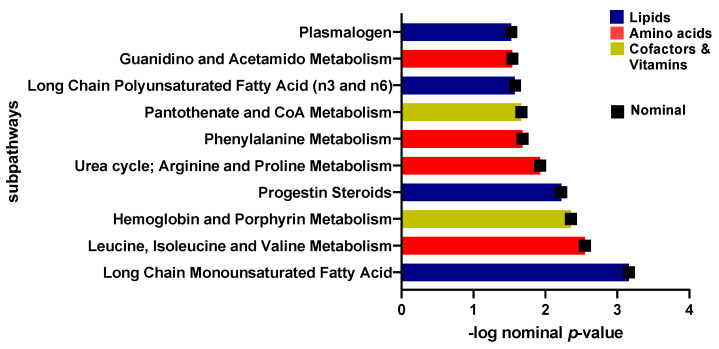
Bar-plot of functional enrichment analysis results based on metabolite ranks by *p*-value using the Wilcoxon rank sum test.

A linear model was used to study the connection between metabolites and sedentary individuals with insulin sensitivity and resistance, controlling for factors such as gender, age, and BMI. Five metabolites were identified to be related to insulin sensitivity and resistance among sedentary individuals and the relevant pathways listed (Table 2). These included pyruvate and glucose (carbohydrate pathway), 1-carboxyethylphenylalanine and N-acetylglycine (amino acid pathway), and gamma-glutamylcitrulline (peptide pathway) (FDR = 0.01). Boxplots of the metabolites with significant differences are shown in Figure 2 and a bar-plot of the results of functional enrichment analysis based on metabolite ranks by p-value using the Wilcoxon rank sum test is shown in Figure 3 and results are indicated in Supplementary Table S2. Enriched metabolic pathways included phenylalanine, plasmalogen, guanidino and acetamido, long chain polyunsaturated fatty acids (n3 and n6), pantothenate and CoA metabolism, urea cycle, arginine and proline, progestin steroids, hemoglobin and porphyrin, leucine, isoleucine and valine, and long chain monounsaturated fatty acids. Heatmap showing the top 100 metabolites is shown in Supplementary Figure S2. Volcano plot (Supplementary Figure S4a depicts log2 fold change and −log10 *p*-value of metabolites that differentiate between IS and IR groups in sedentary individuals. A scatter plot of top metabolites from univariate model are shown in Supplementary Figure S5a.

### 2.4. Univariate Analysis of Metabolites Differentiating Physically Active Insulin Sensitive and Insulin Resistant Individuals

**Figure 4:**
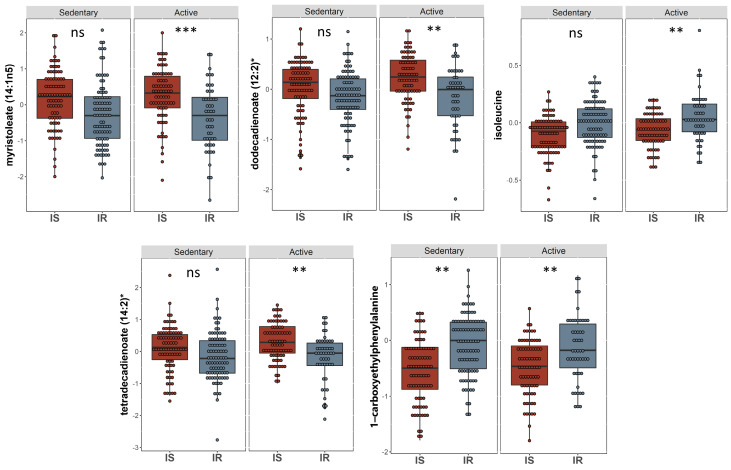
Boxplots of the top hits from the linear regression and functional enrichment analysis from insulin sensitive and insulin resistant individuals who are physically active. Boxplots representing the metabolites with significantly different levels (FDR ≤ 0.05), ***/**/* signifies <0.001/<0.01/<0.05 after adjusting for multiple correction using FDR method. Y-axis indicates level of metabolites in log_e_ scale.

**Figure 5:**
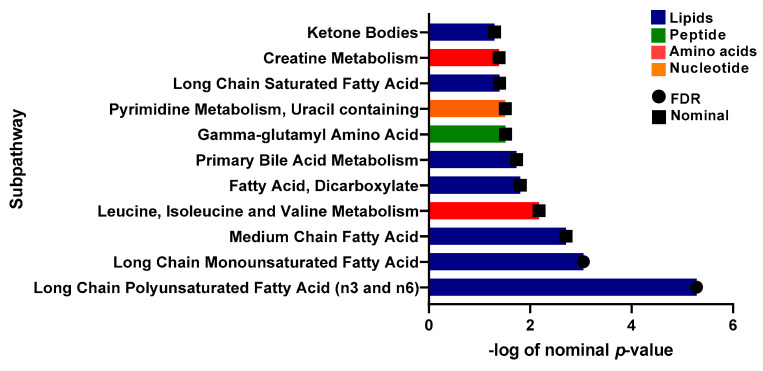
Bar-plot of functional enrichment analysis results based on metabolite ranks by *p*-value using the Wilcoxon rank sum test.

A linear model was applied to evaluate the effect of physical activity on insulin sensitive and resistant individuals by correcting for gender, age, BMI, and PCs. 17 metabolites with significant associations were identified (Table 3). These included 1-carboxyethylphenylalanine and isoleucine (amino acid pathway, FDR = 0.006, 0.007), prolylglycine and gamma-glutamylmethionine (peptide pathway, FDR = 0.031, 0.041), various lipids (FDR < 0.05), and branched-chain, straight-chain, or cyclopropyl 12:1 fatty acid (FDR = 0.006). The metabolites that showed significant differences are illustrated in boxplots in Figure 4. A bar plot summarizing functional enrichment analysis results based on the ranking of metabolites by *p*-value using Wilcoxon rank sum test is shown in Figure 5. Results of functional enrichment analysis indicated significant differences in long chain saturated fatty acids, gamma glutamyl amino acids, fatty acid dicarboxylates, and the metabolism of leucine, isoleucine, valine, medium chain fatty acids, long chain monounsaturated fatty acids, and long chain polyunsaturated fatty acids (n3 and n6) subpathways, as shown in Figure 5 and Supplementary Table S3. Heatmap showing the top 100 metabolites is shown in Supplementary Figure S3. Volcano plot (Supplementary Figure S4b depicts log2 fold change and −log10 *p*-value of metabolites that differentiate between IS and IR groups in active individuals. A scatter plot of top metabolites from univariate model are shown in Supplementary Figure S5b.

### 2.5. Common Metabolites That Are Significantly Different between IS and IR Individuals in Active and Sedentary Groups

**Figure 6:**
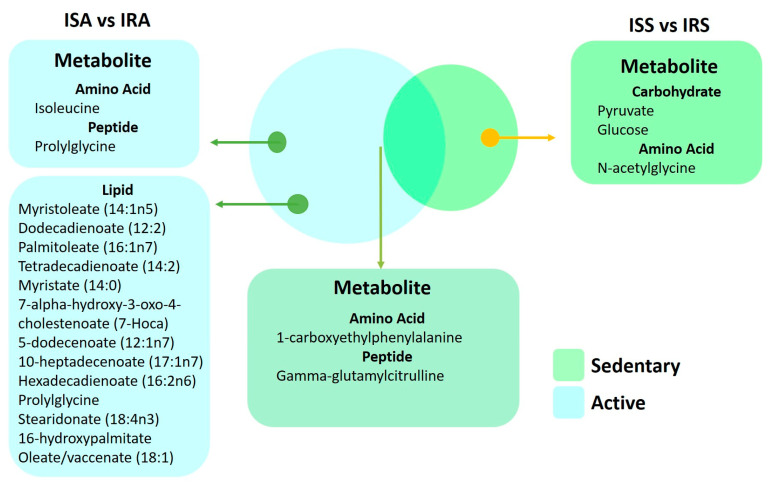
Venn diagram of metabolites from the linear regression showcased in both sedentary and active individuals.

A Venn diagram (Figure 6) illustrates metabolites that are significantly different between IS and IR individuals in active and sedentary groups and those that are common between the two groups. The metabolites 1-carboxyethylphenylalanine and gamma-glutamylcitrulline are associated with insulin sensitive and insulin resistance regardless of activity. Fatty acids(monohydroxy, dicarboxylate, medium and long chain mono and polyunsaturated) were identified in the active groups, whereas changes in carbohydrates (glucose and pyruvate) were seen in the sedentary group.

### 2.6. Spearman’s Correlation of Clinical Traits and Top Metabolite Hits from the Linear Regression Analysis in Active and Sedentary IR Individuals

**Figure 7:**
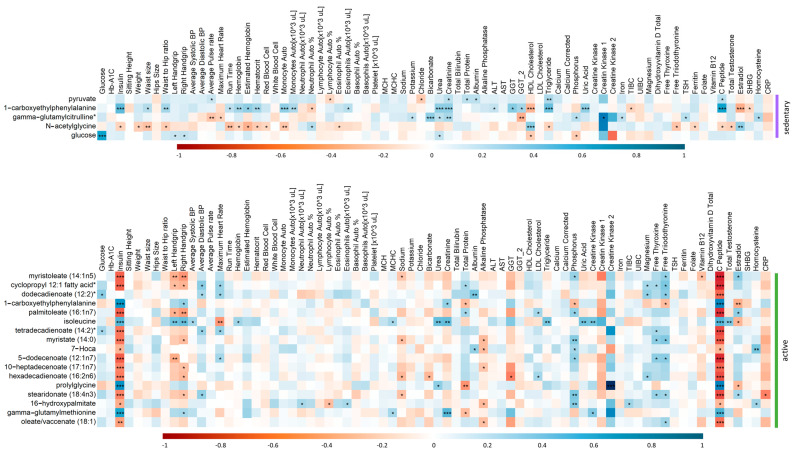
Correlation matrix showing the positive/negative (blue/red) correlation between the metabolites and clinical traits of IR participants in sedentary (top) and active (bottom) groups. Significant correlations are depicted by ***/**/* denoting <0.001/<0.01/<0.05.

In order to further understand how the metabolic changes associated with physical activity are affecting IR individuals, correlation analysis was performed between the clinical traits of IR-sedentary individuals with the significantly changing metabolites from the linear regression analysis (Table 2). Similarly, the clinical traits of IR-active participants were assessed for correlation with metabolites from the linear analysis (Table 3), Figure 7 shows clear significant differences in the correlations of various clinical traits and metabolites differentiating IR active and sedentary individuals. These include negative correlations of insulin and c-peptide and metabolites differentiating IR active, but positive correlation with metabolites differentiating IR sedentary. Conversely, free thyroxine and triiodothyronine are positively correlated with metabolites in IR active, but no correlation in the sedentary group.

## 3. Discussion

The pathology of insulin resistance prior to obesity and its underlying molecular mediators remains largely uncharacterized. Metabolic profiling has been widely used for the identification of novel pathways and specific biomarkers for insulin resistance and T2D [32,33,34,35]. The objective of this study was to compare the metabolic profiling of apparently healthy lean/overweight active and sedentary IS and IR participants and identify the metabolic pathways underpinning the increased risk of the insulin resistance and the impact of physical activity on insulin resistance.

Our emerging data showed 4 distinct metabolic signatures that effectively distinguished between IS and IR, namely glucose and pyruvate in the sedentary group, various types of fatty acids (monohydroxy, dicarboxylate, and short to long chain mono and polyunsaturated) in the active group, and 1-carboxyethylphenylalanine in both groups as a shared microbiota metabolite. Glucose and pyruvate are important molecules in the regulation of insulin sensitivity. Glucose is the primary source of energy for cells and is taken up by cells in response to insulin. Pyruvate is involved in glucose metabolism and is also taken up by cells in response to insulin [36]. In sedentary IR individuals, cells become less sensitive to insulin, resulting in a decreased uptake of glucose and pyruvate. This leads to increased levels of glucose and pyruvate in the bloodstream, which can lead to further progression of disease. This is in line with our results where glucose and pyruvate are higher in IRS. However, in the active group, our results identified several long- and medium-chain fatty acids (PUFA, MUFA, and saturated fatty acids) in active IS & IR but data suggested higher levels of these metabolites in ISA individuals compared to their IRA counterparts. Exercise training promotes fatty acid oxidation in association with suppression of glucose oxidation in skeletal muscle under resting conditions, but increases the rate of carbohydrate oxidation when glucose flux into muscle cells is stimulated by insulin [37].

The transient carbohydrate deficit after exercise caused by the increased demand for energy during exercise results in the mobilization of fatty acids from adipose tissue to provide energy for the muscles. Fat is an important source of energy for muscle contraction, both at rest and during exercise. Triglycerides are stored in adipose tissue and within muscle fibers, and are the main source of the free fatty acids (FFAs) that are oxidized during low intensity exercise [38,39]. At this exercise level, oxidative muscle fibers primarily use fatty acid oxidation for ATP production [40]. The rate of FFA turnover is high enough to provide the majority of the energy needed for exercise. Studies showed that during prolonged exercise, muscle triglycerides become the primary source of energy obtained from fat. Additionally, it is widely documented that endurance activities increase the energy utilization from fat while sparing carbohydrate sources [38,39]. The increased FFA concentration in the plasma was then used to replenish the depleted glycogen stores in the muscles [41]. Our emerging data showed increased levels of long-chain fatty acids in ISA group, suggesting that lipid oxidation in this cohort of young apparently healthy is indeed proportional to lipid availability. In addition to that, emerging findings suggest metabolic switch from glucose to fatty acid metabolism when comparing IS and IR in sedentary vs active individuals. This shift moves metabolism away from lipid/cholesterol synthesis and fat storage, towards the mobilization of fat through fatty acid oxidation in active IS & IR, but higher in ISA. This process helps to maintain muscle mass and function.

Additionally, the emerging data indicate accumulation of various microbiota byproducts such as the phenylalanine derivative 1-carboxyethylphenylalanine in both sedentary & active IR group. 1-carboxyethylphenylalanine was identified as the most discriminating metabolite among all measured metabolites and a promising predictive biomarker for IR [42]. Elevation of phenylalanine and its derivatives (1-carboxyethylphenylalanine) could contribute to progression of insulin resistance as phenylalanine stimulates insulin secretion, potentially causing hyperinsulinemia [43,44]. More recent evidence suggested that phenylalanine impairs insulin signaling and inhibits glucose uptake through modification of insulin receptor (IR) β [45]. Phenylalanine and 1-carboxyethylphenylalanine were found significantly higher in severe insulin resistant (SIRD) [46].

Our data also showed elevation of isoleucine metabolite in IRA compared to ISA, perhaps due to dietary intake. A previous study provides evidence that reducing dietary isoleucine may be an effective way to reduce the risk of insulin resistance. The study also suggests that further research is needed to determine the optimal levels of dietary isoleucine for optimal metabolic health [47]. 

The emerging data also suggested significant negative correlations between 7 lipid metabolites including Myristoleate (14:1n5), Branched-chain, straight-chain, or cyclopropyl 12:1 fatty acid, Dodecadienoate (12:2), Myristate (14:0), 5-dodecenoate (12:1n7), Stearidonate (18:4n3), Oleate/vaccenate (18:1) with insulin and c-peptide in IRA, whereas positive correlations were found with phosphorus, T3 and T4.

The association of top metabolites differencing IS and IR in IRA with insulin and C-peptide is expected, as they are markers of insulin resistance. Previous reports have suggested that long-chain fatty acids exhibit a strong inverse correlation with IS index in a nondiabetic cohort [42]. Additionally, our results suggested that thyroid hormones play a role in lipid metabolism and IS index. A recent study showed that serum thyroid hormone levels have a close correlation with blood lipid and insulin metabolism. The levels of TC, TG, and LDL-C were significantly lower in the hypothyroidism group than in the normal thyroid group [48], suggesting a positive correlation between T3/T4 lipid metabolites, as our results showed in IRA individuals. Another study showed that hypothyroidism is frequently associated with dyslipidemia that results in intrahepatic accumulation of fat, leading to nonalcoholic fatty liver disease (NAFLD), which leads to the development of hepatic insulin resistance. Interestingly, 1-carboxyethylphenylalanine, the common metabolites differentiating IS and IR in in both active and sedentary subjects, was negatively correlated with T3, T4, estradiol, phosphorus, total protein and positively correlated with insulin, creatinine in IRA. Additionally, 1-carboxyethylphenylalanine was positively correlated with insulin, haemoglobin, urea, creatinine, ALT, GGT, GGT2, TG, uric acid and C-peptide in IRS. These correlations provide additional evidence of the potential utility of this metabolite as a surrogate marker of insulin resistance and its associated biochemical markers. The functional relevance of these correlations is currently being investigated.

The study recognizes the limitation of focusing solely on non-obese individuals with insulin resistance and highlights the need for future research to investigate metabolic differences in a more diverse population. Our previous findings on differences between obese IS and IR individuals suggested that phospholipid metabolites are critical in distinguishing between the two groups [34]. The findings of the two studies emphasize the importance of considering physical activity levels and obesity when investigating metabolic differences associated with insulin resistance. Personalized and targeted treatment approaches based on individual metabolic profiles are necessary for effective management of metabolic disorders. Further research is needed to explore the potential clinical implications of these findings and develop effective personalized treatment strategies. Although the study provides valuable insights, there is still much to learn about the complex interplay between metabolism and disease.

## 4. Materials and Methods

### 4.1. The Data Source and Study Participants

The data from 305 participants was extracted from Qatar Biobank, including questionnaires related to physical activity and laboratory results for 66 clinically-relevant metabolic traits such as measurements of systolic and diastolic blood pressure, waist to hip ratio (WHR), body mass index (BMI), clinical chemistry, and endocrinology tests (Table 1). In addition to metabolomics data for over 1000 metabolites. The study was approved by the Institutional Review Boards of the Qatar Biobank (QF-QBB-RES-ACC-00066) and Qatar University (QU-IRB 1716-E/22). All participants provided informed consent. Insulin resistance was determined by HOMA-IR ((fasting glucose (mmol/L) × fasting insulin mlU/mL)/22.5)). Individuals with HOMA-IR less or equal to 1.85 were categorized as IS whereas those with HOMA-IR greater than 1.85 were categorized as IR. Physically active participants were identified as those who walk at least two days per week for more than 150 min. Inclusions criteria included young (20–30 years old) lean/overweight (BMI: 20–30 Kg/m^2^) healthy (no chronic diseases such as diabetes, glaucoma, macular degeneration, blood clot, cardiovascular disease, bariatric surgery, and cancer). Accordingly, among all participants, 42% were physically active, including 25.6% IS (ISA) and 16.4% IR (IRA), whereas 58% were sedentary, including 28.5% IS (ISS) and 29.5% IR (IRS) (Supplementary Figure S1).

### 4.2. Metabolomics

Established protocols were used for untargeted metabolomics of serum samples from all participants [31]. Waters ACQUITY ultra-performance liquid chromatography (UPLC) and a Thermo Scientific Q-Exactive high resolution/accurate mass spectrometer interfaced with a heated electrospray ionization (HESI-II) source and Orbitrap mass analyzer operated at 35,000 mass resolution were used for metabolite measurement. Detailed description of methodology was previously described [31]. To identify compounds, hits were compared with already existing library entries of purified standards of over 3300 purified standard compounds. Compounds were then assigned to various categories according to their sources. Internal standards and quality controls were previously described [49]. Briefly, a mixture of stable isotope-labeled compounds were used as internal standards to correct for variations in sample preparation and instrument performance. Quality control samples were used to monitor the stability and reproducibility of the method over time. The pre-analytical sample handling, including sample collection, storage, and preparation used a standardized protocol to minimize variability and ensure the integrity of the samples. Supplementary Table S4 lists all identified metabolites and their raw data.

### 4.3. Statistical Analysis

The metabolomics data were log-transformed. Multivariate analysis including unsupervised (principle component analysis) PCA and supervised (orthogonal partial least square-discriminant analysis) OPLS-DA were run using the software SIMCA^®^ (version 16.0.1). Two outliers were removed after PCA analysis as a part of quality control prior to OPLS-DA. R version 4.0.3 was used to perform linear models for each metabolite (as the response variable) versus insulin sensitivity (HOMA-IR cutoff of 1.85), physical activity (active/sedentary) (as the explanatory variables) and their interaction. The model also included the following confounders: age, gender, and BMI. Marginal means were compared between insulin-sensitive (IS) (HOMA-IR < 1.85) and insulin-resistant (IR) (HOMA-IR > 1.85) individuals stratified by physical activity status using the R package-Emmeans. Multiple testing correction method (False Discovery Rate, FDR) was used to adjust the nominal *p*-values. FDR < 0.05 was considered statistically significant. Functional enrichment analysis was run on all *p*-value ordered metabolite lists from linear models performed in the study. This analysis was conducted based on one-way Wilcoxon rank sum test which was followed by FDR multiple testing correction method. The sub pathways were previously predefined by Metabolon and those with less than three top hits were dropped. Spearman’s correlation was performed between the significantly different metabolites from the regression analysis and the clinical measurements of participants for IRA and ISA groups separately. A *p* value of 0.05 was considered significant.

## 5. Conclusions

This study investigated the metabolic signatures of IS and IR non-obese individuals, both active and sedentary. Findings revealed that physical activity influences the metabolic pathways of IR individuals by shifting their metabolism from glucose-based to fat-based. These insights highlight the importance of moderate physical activity in reducing metabolic syndrome risk in non-obese individuals, offering crucial implications for prevention and treatment strategies.

## Figures and Tables

**Table 1:** General characteristics of study participants.

	Physically Active			Sedentary		
	ISA (78)	IRA (50)	*p*	ISS (87)	IRS (90)	*p*
**Gender**						
Male	44 (56.41%)	26 (52%)	0.624	33 (37.93%)	56 (62.23%)	0.001
Female	34 (43.58%)	24 (48%)		54 (62.06%)	34 (37.78%)	
**Vital Signs**						
Age (years)	25.7 (2.7)	26.0 (2.9)	0.562	25.8 (3.0)	25.8 (3.0)	0.881
BMI (kg/m^2^)	24.4 (22.9–26.9)	26.1 (23.4–27.8)	0.174	23.7 (22.2–26.0)	24.9 (23.4–27.5)	1.3 × 10^−3^
Average systolic BP (mmHg)	106.0 (101.0–113.7)	108.5 (101.0–116.0)	0.355	102.0 (96.0–111.0)	110.0 (101.0–115.7)	2.3 × 10^−3^
Average diastolic BP (mmHg)	67.8 (7.4)	67.4 (7.6)	0.957	67.5 (8.1)	69.74 (7.30)	0.043
Pulse rate (beats/min)	65.0 (59.0–72.0)	69.0 (63.0–76.0)	0.062	68.0 (62.0–72.0)	70.0 (65.0–78.0)	0.048
**Blood sugar**						
HOMA-IR	1.2 (1.0–1.5)	2.9 (2.3–5.0)	1.7 × 10^−21^	1.3 (1.0–1.5)	3.2 (2.2–5.2)	3.2 × 10^−30^
C Peptide (ng/mL)	1.4 (1.2–1.7)	2.6 (2.1–4.0)	9.6 × 10^−16^	1.5 (1.3–1.7)	2.9 (2.1–4.2)	4.8 × 10^−18^
Insulin (uu/mL)	6.0 (4.9–7.0)	13.2 (10.2–21.9)	1.0 × 10^−20^	6.0 (4.8–7.0)	14.5 (10.0–23.1)	6.9 × 10^−27^
Hemoglobin A1c (HbA1c) %	5.2 (5.0–5.3)	5.3 (5.0–5.4)	0.182	5.2 (5.0–5.4)	5.3 (5.1–5.5)	0.124
Glucose (mmol/L)	4.8 (4.5–5.0)	5.1 (4.8–5.4)	1.1 × 10^−4^	4.7 (4.5–4.9)	5.2 (4.8–5.4)	6.2 × 10^−11^
**Physical tests**						
Sitting height (cm)	90.7 (85.2–134.3)	90.8 (85.5–134.1)	0.778	91.7 (86.4–134.8)	91.4 (87.2–133.6)	0.783
Weight (kg)	69.2 (61.3–76.2)	73.4 (63.2–79.8)	0.134	65.4 (58.6–72.2)	70.4 (63.9–78.7)	2.1 × 10^−3^
Waist size (cm)	78.5 (73.0–84.0)	82.5 (75.0–89.0)	0.044	75.0 (71.0–80.0)	82.0 (75.0–89.0)	4.0 × 10^−6^
Hip size (cm)	100.2 ± 6.8	102.3 ± 6.1	0.072	100.5 ± 6.8	101.1 ± 5.8	0.463
Waist to hip ratio	0.79 ± 0.07	0.80 ± 0.08	0.305	0.76 ± 0.08	0.81 ± 0.08	2.2 × 10^−5^
Handgrip (left)	33.0 (22.0–44.0)	33.0 (28.0–43.0)	0.925	26.0 (20.0–38.0)	31.5 (22.0–40.0)	0.046
Handgrip (right)	35.0 (26.0–48.0)	32.0 (26.0–45.5)	0.739	29.0 (22.0–39.5)	34.0 (23.2–42.0)	0.114
Maximum heart rate (beats/min)	120.0 (106.5–133.0)	122.0 (111.5–132.0)	0.485	129.0 (112.0–142.0)	126.5 (114.2–139.0)	0.893
Run time (seconds)	764.0 (742.0–764.0)	764.0 (742.0–764.0)	0.981	764.0 (684.0–764.0)	764.0 (742.0–764.0)	0.12
**Kidney profile**						
Sodium (mmol/L)	140.5 (139.0–141.8)	141.0 (139.0–142.0)	0.739	140.0 (138.5–141.0)	140.0 (139.0–142.0)	0.133
Potassium (mmol/L)	4.3 (4.1–4.5)	4.3 (4.1–4.5)	0.787	4.3 (4.1–4.5)	4.2 (4.0–4.4)	0.037
Chloride (mmol/L)	101.0 (100.0–102.0)	102.0 (99.2–102.7)	0.398	101.0 (100.0–102.0)	101.0 (100.0–102.0)	0.794
Bicarbonate (mmol/L)	27.0 (25.0–28.0)	26.0 (25.2–28.0)	0.41	26.0 (25.0–27.0)	26.0 (25.0–27.0)	0.826
Urea (mmol/L)	4.3 (3.4–5.3)	4.1 (3.4–5.1)	0.606	4.2 (3.3–4.9)	4.2 (3.4–4.8)	0.822
Creatinine (mmol/L)	69.0 (52.7–78.7)	67.0 (54.7–80.0)	0.743	61.0 (54.0–76.5)	70.5 (58.2–77.0)	0.023
Calcium (mmol/L)	2.4 ± 0.1	2.4 ± 0.1	0.571	2.4 ± 0.1	2.4 ± 0.1	0.264
Calcium Corrected (mmol/L)	2.3 ± 0.1	2.3 ± 0.1	0.382	2.3 ± 0.1	2.3 ± 0.1	0.146
Phosphorus (mmol/L)	1.2 ± 0.1	1.2 ± 0.2	0.752	1.2 ± 0.1	1.1 ± 0.2	1.1 × 10^−3^
Uric Acid (umol/L)	296.5 (240.2–335.5)	282.0 (234.5–361.7)	0.625	257.0 (221.5–305.0)	308.5 (242.0–349.7)	6.1 × 10^−4^
Creatine kinase (U/L)	86.5 (69.2–130.0)	99.0 (60.0–159.5)	0.746	67.0 (55.0–105.0)	89.0 (62.0–117.0)	0.037
Creatine Kinase 1 (ng/mL)	1.1 (0.9–1.5)	1.1 (0.7–1.5)	0.771	1.0 (0.7–2.0)	1.31 (1.0–1.6)	1
Creatine kinase 2 (U/L)	88.0 (72.0–124.0)	71.0 (52.2–239.0)	0.758	86.5 (60.0–127.0)	75 (47.5–109)	0.478
Magnesium(µmol/L)	0.8 ± 0.05	0.8 ± 0.05	0.887	0.8 ± 0.05	0.84 ± 0.05	0.621
Total Protein (g/L)	74.0 (72.0–76.0)	75.0 (72.0–77.0)	0.737	73.0 (70.0–76.0)	74.0 (72.0–76.0)	0.049
Homocysteine (µmol/L)	8.7 (6.8–10.6)	8.0 (6.6–9.5)	0.142	8.3 (6.2–9.9)	8.0 (6.6–9.5)	0.952
**Liver profile**						
Total Bilirubin (µmol/L)	7.0 (5.2–10.0)	5.9 (4.6–7.85)	0.045	7.6 (6.0–9.0)	6.0 (4.3–9.0)	0.031
Albumin (g/L)	47.0 (45.0–48.0)	47.0 (45.0–48.0)	0.63	46.0 (45.0–48.0)	46.0 (44.2–49.0)	0.992
Alkaline phosphatase (U/L)	61.0 (48.5–72.0)	64.5 (57.2–79.7)	0.054	60.0 (52.5–70.5)	68.5 (60.0–77.7)	1.1 × 10^−3^
Alanine Transaminase (U/L)	17.0 (12.0–22.7)	19.0 (13.0–31.5)	0.292	14.0 (10.0–19.5)	21.5 (14.0–32.0)	8.5 × 10^−6^
Aspartate aminotransferase (U/L)	19.0 (16.0–21.7)	18.0 (15.0–23.7)	0.835	17.0 (14.0–19.0)	18.5 (15.0–21.0)	8.3 × 10^−3^
GGT (U/L)	13.0 (10.5–18.5)	13.0 (10.5–17.5)	0.788	13.0 (8.2–17.0)	16.0 (13.0–23.0)	0.037
GGT 2 (U/L)	14.0 (10.0–19.0)	17.5 (12–25.5)	0.069	14.0 (12.0–25.0)	23.0 (16.0–35.0)	2.7 × 10^−4^
**Lipid profile**						
HDL Cholesterol (mmol/L)	1.5 ± 0.4	1.4 ± 0.4	0.356	1.5 ± 0.3	1.2 ± 0.3	1.5 × 10^−5^
LDL Cholesterol Calc (mmol/L)	2.8 (2.1–3.0)	2.9 (2.1–3.3)	0.413	2.6 (2.1–3.0)	2.9 (2.5–3.2)	0.082
Triglyceride (mmol/L)	0.8 (0.6–1.0)	1.0 (0.7–1.4)	0.041	0.8 (0.5–1.1)	1.1 (0.8–1.6)	3.7 × 10^−6^
**Iron profile**						
Iron (µmol/L)	15.5 (11.5–20.0)	14.0 (10.5–17.0)	0.143	16.0 (10.5–20.0)	15.15 (11.21–19.1)	0.971
TIBC (µmol/L)	56.0 (52.0–63.0)	60.0 (56.0–67.0)	0.004	60.5 (54.2–66.5)	59.0 (52.0–64.0)	0.443
UIBC (µmol/L)	41.0 (34.0–48.0)	46.0 (39.0–55.0)	0.014	43.0 (36.0–53.6)	42.0 (34.5–49.8)	0.583
Ferritin (µg/L)	54.0 (22.0–110.5)	34.0 (17.0–105.5)	0.332	34.0 (10.0–70.0)	63.5 (20.5–119.0)	0.014
**Vitamins**						
Folate (nmol/L)	22.0 ± 7.4	22.6 ± 8.2	0.673	24.0 ± 8.9	21.6 ± 7.0	0.157
Vitamin B12 (pmol/L)	288.0 (232.2–418.0)	271.5 (208.0–356.2)	0.194	283.0 (222.0–374.0)	295.0 (229.0–377.0)	0.935
Dihydroxyvitamin D Total (ng/mL)	15.0 (11.2–18.0)	13.5 (11.0–24.0)	0.46	16.5 (12.0–24.7)	13.0 (10.2–17.7)	4.2 × 10^3^
**Hormones**						
Total Testosterone (nmol/L)	15.9 (1.4–25.4)	4.14 (1.2–17.6)	0.047	1.85 (1.1–20.4)	14.2 (1.4–19.0)	0.383
Estradiol (pmol/L)	124.0 (88.0–237.0)	122.0 (84.0–234.0)	0.499	146 (86.7–349.7)	105 (77.2–169.7)	0.022
SHBG (nmol/L)	44.3 (30.8–64.7)	37.1 (23.5–60.0)	0.1	52.8 (36.7–79.2)	31.5 (22.5–46.8)	4.1 × 10^−6^
Free Thyroxine (pmol/L)	13.7 (12.5–14.5)	13.3 (12.5–14.1)	0.469	13.3 (12.4–14.6)	13.4 (12.6–14.6)	0.63
Free triiodothyronine (nmol/L)	4.6 (4.1–4.9)	4.4 (4.0–4.85)	0.701	4.4 (4.14–4.8)	4.7 (4.3–5.1)	0.007
TSH (mU/L)	1.3 (1.0–2.0)	1.3 (1.0–1.6)	0.241	1.6 (1.1–2.5)	1.3 (0.9–2.2)	0.079
**Blood inflammatory markers**						
Hemoglobin (g/dL)	13.7 (12.6–15.1)	13.7 (12.5–14.9)	0.655	13.3 (11.9–14.56)	14.4 (13.1–15.3)	8.6 × 10^−4^
Estimated hemoglobin *	18.5 ± 3.3	19.1 ± 3.3	0.527	17.7 ± 3.8	18.3 ± 3.9	0.236
Hematocrit %	42.1 (37.6–45.0)	41.9 (38.5–44.9)	0.843	40.0 (36.1–42.78)	42.7 (39.4–45.7)	4.2 × 10^−4^
Red Blood Cell count (×10^6^/µL)	5 (4.4–5.3)	5.1 (4.6–5.5)	0.095	4.7 (4.5–5.1)	5.1 (4.7–5.6)	3.5 × 10^−5^
White Blood Cell count (×10^3^/µL)	6.5 (5.4–7.5)	6.5 (5.5–8.1)	0.696	6.4 (5.4–7.6)	6.6 (5.1–7.8)	0.984
Monocyte %	7 (5.9–8.2)	7.3 (6.2–8.6)	0.506	6.8 (5.6–8.2)	8.0 (6.5–9.3)	2.8 x 10^−3^
Monocyte count (×10^3^/µL)	0.5 (0.4–0.6)	0.5 (0.4–0.6)	0.473	0.4 (0.3–0.5)	0.5 (0.4–0.6)	8.7 × 10^−4^
Absolute neutrophil count (×10^3^/µL)	3.3 (2.7–4.3)	3.6 (2.8–4.5)	0.535	3.4 (2.7–4.7)	3.5 (2.6–4.4)	0.901
Neutrophil %	53.2 ± 9.6	55.0 ± 10.3	0.325	54.2 ± 9.0	54.6 ± 9.6	0.847
Lymphocyte count (×10^3^/µL)	2.3 (1.9–2.8)	2.1 (1.9–2.5)	0.386	2.2 (1.8–2.6)	2.2 (1.7–2.6)	0.477
Lymphocyte %	36.5 ± 8.7	34.1 ± 8.7	0.134	35.6 ± 7.6	34.4 ± 7.8	0.291
Eosinophil %	2.3 (1.3–3.8)	2.3 (1.5–3.475)	0.966	1.8 (1.2–3.3)	2.5 (1.6–3.5)	0.049
Eosinophils count (×10^3^/µL)	0.1 (0.1–0.2)	0.1 (0.1–0.3)	0.911	0.1 (0.1–0.2)	0.1 (0.1–0.2)	0.327
Basophil %	0.6 (0.3–0.7)	0.6 (0.4–0.7)	0.601	0.7 (0.5–0.9)	0.6 (0.4–0.8)	0.231
Basophils count (×10^3^/µL)	0.0 (0.0–0.1)	0.0 (0.0–0.03)	0.864	0.0 (0.0–0.1)	0.0 (0.0–0.1)	0.589
Platelet count (×10^3^/µL)	233 (207.0–263.0)	235 (208.8–275.8)	0.674	237 (195.0–284.0)	244 (209.0–276.5)	0.805
MCH (pg)	28.6 (26.6–29.9)	27.5 (25.7–28.8)	0.013	28.6 (26.1–29.8)	28.2 (26.6–29.6)	0.743
MCHC (g/dL)	33.4 (32.7–33.9)	33 (32.4–33.6)	0.184	33.3 (32.5–34.0)	33.6 (32.8–34.2)	0.185
C Reactive Protein (mg/L)	5 (5–5)	5 (5–5)	0.659	5 (5–5)	5 (5–5)	0.023

BMI (body mass index), Systolic BP (systolic blood pressure), Diastolic BP (diastolic blood pressure), LDL (low density lipoprotein), HDL (high density lipoprotein), HOMA-IR (homeostatic model assessment of insulin resistance), TIBC (Total iron binding capacity), UIBC (Unsaturated iron binding capacity), SHBG (Sex hormone binding globulin), TSH (Thyroid stimulating hormone), MCH (Mean cell hemoglobin), MCHC (Mean corpuscular hemoglobin concentration), * hemoglobin (estimated from hematocrit). The data is presented as mean ± SD for normal variables, median (IQR) for skewed variables, and number (percentage) for nominal parameters. The differences between the groups were tested using ANOVA/Kruskal Wallis for parametric/non-parametric variables and Chi-square test for nominal variables. The significance level was set at *p* ≤ 0.05.

**Table 2:** Linear model analysis of metabolite levels in insulin sensitive versus insulin resistant amongst sedentary individuals.

Metabolite	Super-Pathway	Sub-Pathway	Estimate	SE	*p*-Value	FDR
Pyruvate	Carbohydrate	Glycolysis, Gluconeogenesis, and Pyruvate Metabolism	−0.182	0.033	7.22 × 10^−8^	6.11 × 10^−5^
1-carboxyethylphenylalanine	Amino Acid	Phenylalanine Metabolism	−0.372	0.079	3.76 × 10^−6^	0.001
Gamma-glutamylcitrulline	Peptide	Gamma-glutamyl Amino Acid	0.214	0.051	3.59 × 10^−5^	0.010
N-acetylglycine	Amino Acid	Glycine, Serine and Threonine Metabolism	0.268	0.068	1.01 × 10^−4^	0.021
Glucose	Carbohydrate	Glycolysis, Gluconeogenesis, and Pyruvate Metabolism	−0.103	0.028	2.31 × 10^−4^	0.039

**Table 3:** Linear model analysis of metabolite levels in insulin sensitive versus insulin resistant amongst active individuals.

Metabolite	Super-Pathway	Sub-Pathway	Estimate	SE	*p*-Value	FDR
Myristoleate (14:1n5)	Lipid	Long Chain Monounsaturated Fatty Acid	0.684	0.138	1.38 × 10^−6^	0.001
Branched-chain, straight-chain, or cyclopropyl 12:1 fatty acid	Partially Characterized Molecules	Partially Characterized Molecules	0.597	0.136	1.59 × 10^−5^	0.006
Dodecadienoate (12:2)	Lipid	Fatty Acid, Dicarboxylate	0.415	0.098	2.67 × 10^−5^	0.006
1-carboxyethylphenylalanine	Amino Acid	Phenylalanine Metabolism	−0.395	0.093	2.90 × 10^−5^	0.006
Palmitoleate (16:1n7)	Lipid	Long Chain Monounsaturated Fatty Acid	0.584	0.141	4.58 × 10^−5^	0.007
Isoleucine	Amino Acid	Leucine, Isoleucine and Valine Metabolism	−0.119	0.029	5.59 × 10^−5^	0.007
Tetradecadienoate (14:2)	Lipid	Long Chain Polyunsaturated Fatty Acid (n3 and n6)	0.499	0.123	6.40 × 10^−5^	0.007
Myristate (14:0)	Lipid	Long Chain Saturated Fatty Acid	0.407	0.102	8.04 × 10^−5^	0.008
7-alpha-hydroxy-3-oxo-4-cholestenoate (7-Hoca)	Lipid	Sterol	0.194	0.05	1.57 × 10^−4^	0.015
5-dodecenoate (12:1n7)	Lipid	Medium Chain Fatty Acid	0.42	0.112	2.16 × 10^−4^	0.017
10-heptadecenoate (17:1n7)	Lipid	Long Chain Monounsaturated Fatty Acid	0.439	0.117	2.24 × 10^−4^	0.017
Hexadecadienoate (16:2n6)	Lipid	Long Chain Polyunsaturated Fatty Acid (n3 and n6)	0.43	0.12	4.21 × 10^−4^	0.029
Prolylglycine	Peptide	Dipeptide	−0.375	0.106	4.87 × 10^−4^	0.031
Stearidonate (18:4n3)	Lipid	Long Chain Polyunsaturated Fatty Acid (n3 and n6)	0.448	0.13	6.55 × 10^−4^	0.036
16-hydroxypalmitate	Lipid	Fatty Acid, Monohydroxy	0.227	0.066	6.61 × 10^−4^	0.036
Gamma-glutamylmethionine	Peptide	Gamma-glutamyl Amino Acid	−0.195	0.057	8.04 × 10^−4^	0.041
Oleate/vaccenate (18:1)	Lipid	Long Chain Monounsaturated Fatty Acid	0.369	0.109	8.29 × 10^−4^	0.041

## Data Availability

The datasets used and/or analysed during the current study are available from the corresponding author on reasonable request.

## Supplementary Materials

The following supporting information can be downloaded at: https://www.mdpi.com/article/10.3390/ijms24097816/s1**.**
