# Supplementary material for: The Metabolic Switch of Physical Activity in Non-Obese Insulin Resistant Individuals
Source: Int J Mol Sci. 2023 Apr 25;24(9):7816. doi: 10.3390/ijms24097816 (PMC10178125; doi:10.3390/ijms24097816)
Supplement: Supplementary file 1 [file ijms-24-07816-s001.zip › Supplementary figures and tables_final.pdf]

Supplementary Figure 1: OPLS-DA of four groups observed 2 principal components with R2Y of 15.2% and R2X of 12.3%.

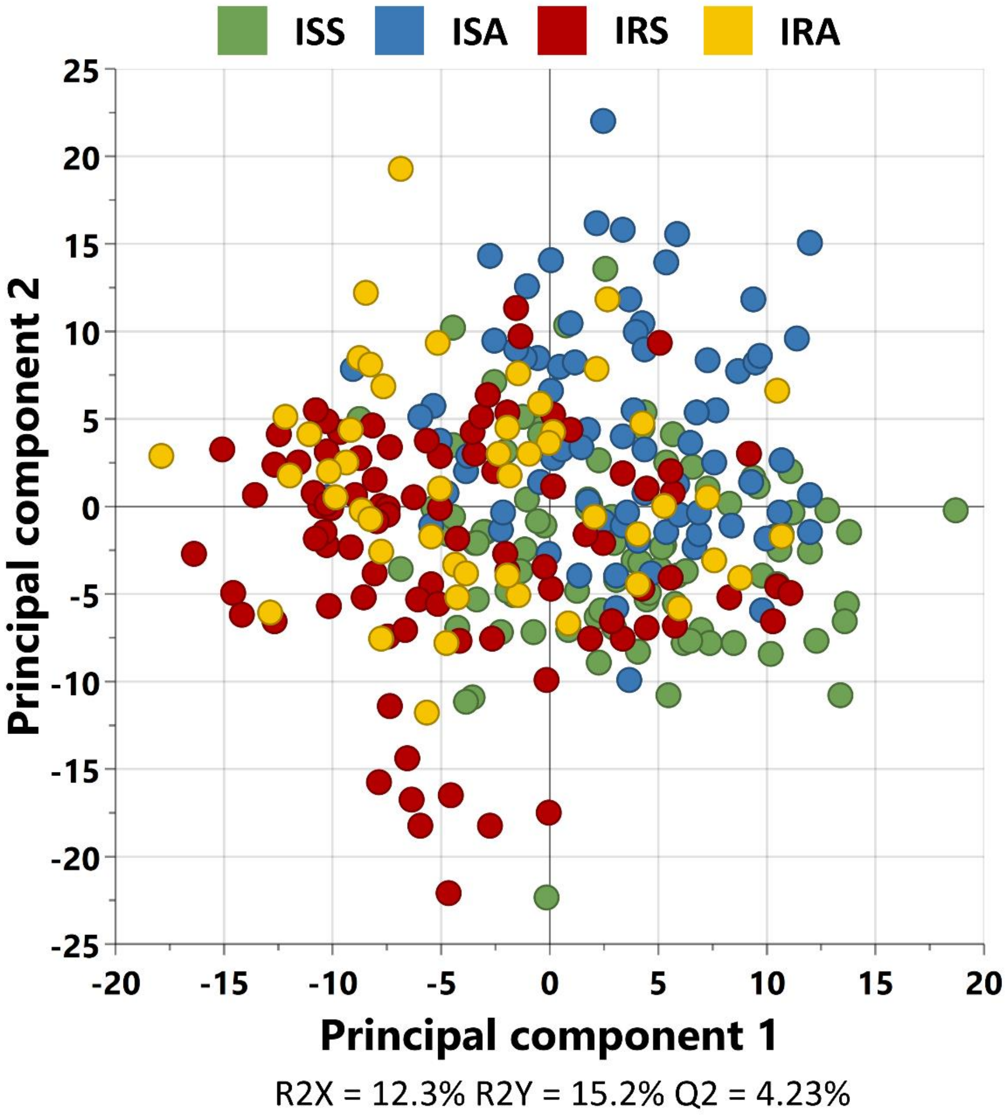

Supplementary table 1: Corresponding Variable importance of projection from OPLS-DA of four groups, metabolites with VIP score > 1.5 are mentioned.

Metabolite	Super pathway	Sub pathway	VIP SCORE
1-carboxyethylphenylalanine	Amino Acid	Phenylalanine Metabolism	2.37306
gamma-glutamylleucine	Peptide	Gamma-glutamyl Amino Acid	2.34239
palmitoleate (16:1n7)	Lipid	Long Chain Monounsaturated Fatty Acid	2.29808
myristoleate (14:1n5)	Lipid	Long Chain Monounsaturated Fatty Acid	2.26711
branched-chain, straight-chain, or cyclopropyl 12:1 fatty acid*	Partially Characterized Molecules	Partially Characterized Molecules	2.24644
gamma-glutamylisoleucine*	Peptide	Gamma-glutamyl Amino Acid	2.20521
leucine	Amino Acid	Leucine, Isoleucine and Valine Metabolism	2.17993
gamma-glutamylvaline	Peptide	Gamma-glutamyl Amino Acid	2.17101
creatinine	Amino Acid	Creatine Metabolism	2.17072
5-dodecenoate (12:1n7)	Lipid	Medium Chain Fatty Acid	2.15556

isoleucine	Amino Acid	Leucine, Isoleucine and Valine Metabolism	2.1298
isovalerylcarnitine (C5)	Amino Acid	Leucine, Isoleucine and Valine Metabolism	2.10341
2-methylbutyrylcarnitine (C5)	Amino Acid	Leucine, Isoleucine and Valine Metabolism	2.09172
tetradecadienoate (14:2)*	Lipid	Long Chain Polyunsaturated Fatty Acid (n3 and n6)	2.06867
picolinoylglycine	Lipid	Fatty Acid Metabolism (Acyl Glycine)	2.06648
oleate/vaccenate (18:1)	Lipid	Long Chain Monounsaturated Fatty Acid	2.06103
10-heptadecenoate (17:1n7)	Lipid	Long Chain Monounsaturated Fatty Acid	2.02649
valine	Amino Acid	Leucine, Isoleucine and Valine Metabolism	2.0263
2-hydroxy-3-methylvalerate	Amino Acid	Leucine, Isoleucine and Valine Metabolism	2.02484
dodecadienoate (12:2)*	Lipid	Fatty Acid, Dicarboxylate	2.0236
N-acetylcarnosine	Amino Acid	Histidine Metabolism	2.02189
phenyllactate (PLA)	Amino Acid	Phenylalanine Metabolism	2.0161
3-(4-hydroxyphenyl)lactate	Amino Acid	Tyrosine Metabolism	2.00173
gamma-glutamylphenylalanine	Peptide	Gamma-glutamyl Amino Acid	1.9869
hexadecadienoate (16:2n6)	Lipid	Long Chain Polyunsaturated Fatty Acid (n3 and n6)	1.97593
myristate (14:0)	Lipid	Long Chain Saturated Fatty Acid	1.94938
3-hydroxybutyrate (BHBA)	Lipid	Ketone Bodies	1.94034
alpha-hydroxyisocaproate	Amino Acid	Leucine, Isoleucine and Valine Metabolism	1.93179
linoleate (18:2n6)	Lipid	Long Chain Polyunsaturated Fatty Acid (n3 and n6)	1.92653
indolelactate	Amino Acid	Tryptophan Metabolism	1.91426
N-acetylglycine	Amino Acid	Glycine, Serine and Threonine Metabolism	1.90245
deoxycarnitine	Lipid	Carnitine Metabolism	1.87486
2-oxoarginine*	Amino Acid	Urea cycle; Arginine and Proline Metabolism	1.86856
10-nonadecenoate (19:1n9)	Lipid	Long Chain Monounsaturated Fatty Acid	1.86784
eicosenoate (20:1)	Lipid	Long Chain Monounsaturated Fatty Acid	1.86545
urate	Nucleotide	Purine Metabolism, (Hypo)Xanthine/Inosine containing	1.85349
hydroxy-N6,N6,N6-trimethyllysine*	Amino Acid	Lysine Metabolism	1.85062
1-methylhistidine	Amino Acid	Histidine Metabolism	1.84485
N,N,N-trimethyl-alanylproline betaine (TMAP)	Amino Acid	Urea cycle; Arginine and Proline Metabolism	1.83996
2-aminoadipate	Amino Acid	Lysine Metabolism	1.83334
(2 or 3)-decenoate (10:1n7 or n8)	Lipid	Medium Chain Fatty Acid	1.81297
gamma-glutamylmethionine	Peptide	Gamma-glutamyl Amino Acid	1.79799
3-methyl-2-oxovalerate	Amino Acid	Leucine, Isoleucine and Valine Metabolism	1.7978
prolylglycine	Peptide	Dipeptide	1.7859
gamma-glutamyltyrosine	Peptide	Gamma-glutamyl Amino Acid	1.77783
tryptophan	Amino Acid	Tryptophan Metabolism	1.77648
N6,N6,N6-trimethyllysine	Amino Acid	Lysine Metabolism	1.77425
N-acetylleucine	Amino Acid	Leucine, Isoleucine and Valine Metabolism	1.77404
4-methyl-2-oxopentanoate	Amino Acid	Leucine, Isoleucine and Valine Metabolism	1.77345
tiglylcarnitine (C5:1-DC)	Amino Acid	Leucine, Isoleucine and Valine Metabolism	1.76315
phenylalanine	Amino Acid	Phenylalanine Metabolism	1.75198
xanthurenate	Amino Acid	Tryptophan Metabolism	1.74869

2,3-dihydroxy-5-methylthio-4-pentenoate (DMTPA)*	Amino Acid	Methionine, Cysteine, SAM and Taurine Metabolism	1.74385
10-undecenoate (11:1n1)	Lipid	Medium Chain Fatty Acid	1.74155
kynurenate	Amino Acid	Tryptophan Metabolism	1.73611
11beta-hydroxyandrosterone glucuronide	Lipid	Androgenic Steroids	1.73092
cis-4-decenoate (10:1n6)*	Lipid	Medium Chain Fatty Acid	1.72952
dihomo-linoleate (20:2n6)	Lipid	Long Chain Polyunsaturated Fatty Acid (n3 and n6)	1.72559
gamma-glutamyl-2-aminobutyrate	Peptide	Gamma-glutamyl Amino Acid	1.71999
alpha-hydroxyisovalerate	Amino Acid	Leucine, Isoleucine and Valine Metabolism	1.71953
linolenate [alpha or gamma; (18:3n3 or 6)]	Lipid	Long Chain Polyunsaturated Fatty Acid (n3 and n6)	1.71575
argininate*	Amino Acid	Urea cycle; Arginine and Proline Metabolism	1.70446
3-hydroxydecanoate	Lipid	Fatty Acid, Monohydroxy	1.69048
cysteinylglycine disulfide*	Amino Acid	Glutathione Metabolism	1.6864
5alpha-androstan-3beta,17beta-diol disulfate	Lipid	Androgenic Steroids	1.68575
palmitate (16:0)	Lipid	Long Chain Saturated Fatty Acid	1.68514
1-carboxyethylvaline	Amino Acid	Leucine, Isoleucine and Valine Metabolism	1.68513
pentadecanoate (15:0)	Lipid	Long Chain Saturated Fatty Acid	1.68049
gamma-glutamylhistidine	Peptide	Gamma-glutamyl Amino Acid	1.67372
S-carboxyethylcysteine	Amino Acid	Methionine, Cysteine, SAM and Taurine Metabolism	1.67287
cys-gly, oxidized	Amino Acid	Glutathione Metabolism	1.66244
docosadienoate (22:2n6)	Lipid	Long Chain Polyunsaturated Fatty Acid (n3 and n6)	1.64032
decanoylcarnitine (C10)	Lipid	Fatty Acid Metabolism (Acyl Carnitine, Medium Chain)	1.62648
3-hydroxy-2-ethylpropionate	Amino Acid	Leucine, Isoleucine and Valine Metabolism	1.62585
4-guanidinobutanoate	Amino Acid	Guanidino and Acetamido Metabolism	1.61437
N-acetylisoleucine	Amino Acid	Leucine, Isoleucine and Valine Metabolism	1.61223
EDTA	Xenobiotics	Chemical	1.60788
kynurenine	Amino Acid	Tryptophan Metabolism	1.60438
isobutyrylcarnitine (C4)	Amino Acid	Leucine, Isoleucine and Valine Metabolism	1.59764
3,5-dichloro-2,6-dihydroxybenzoic acid	Xenobiotics	Chemical	1.59603
picolinate	Amino Acid	Tryptophan Metabolism	1.57207
hexanoylglutamine	Lipid	Fatty Acid Metabolism (Acyl Glutamine)	1.56466
2-aminobutyrate	Amino Acid	Glutathione Metabolism	1.56052
stearidonate (18:4n3)	Lipid	Long Chain Polyunsaturated Fatty Acid (n3 and n6)	1.55328
3-bromo-5-chloro-2,6-dihydroxybenzoic acid*	Xenobiotics	Chemical	1.54977
sarcosine	Amino Acid	Glycine, Serine and Threonine Metabolism	1.54167
N-acetylphenylalanine	Amino Acid	Phenylalanine Metabolism	1.53688
docosapentaenoate (n3 DPA; 22:5n3)	Lipid	Long Chain Polyunsaturated Fatty Acid (n3 and n6)	1.5329
gamma-glutamyl-alpha-lysine	Peptide	Gamma-glutamyl Amino Acid	1.52827
propionylcarnitine (C3)	Lipid	Fatty Acid Metabolism (also BCAA Metabolism)	1.51959
5alpha-androstan-3alpha,17beta-diol disulfate	Lipid	Androgenic Steroids	1.51684
N-acetyltryptophan	Amino Acid	Tryptophan Metabolism	1.51682
1-methylguanidine	Amino Acid	Guanidino and Acetamido Metabolism	1.50709

2-hydroxy-4-(methylthio)butanoic acid	Amino Acid	Methionine, Cysteine, SAM and Taurine Metabolism	1.50547

Supplementary figure 2: Heatmap of top 100 metabolites from the univariate analysis that differentiate IS and IR among sedentary individuals.

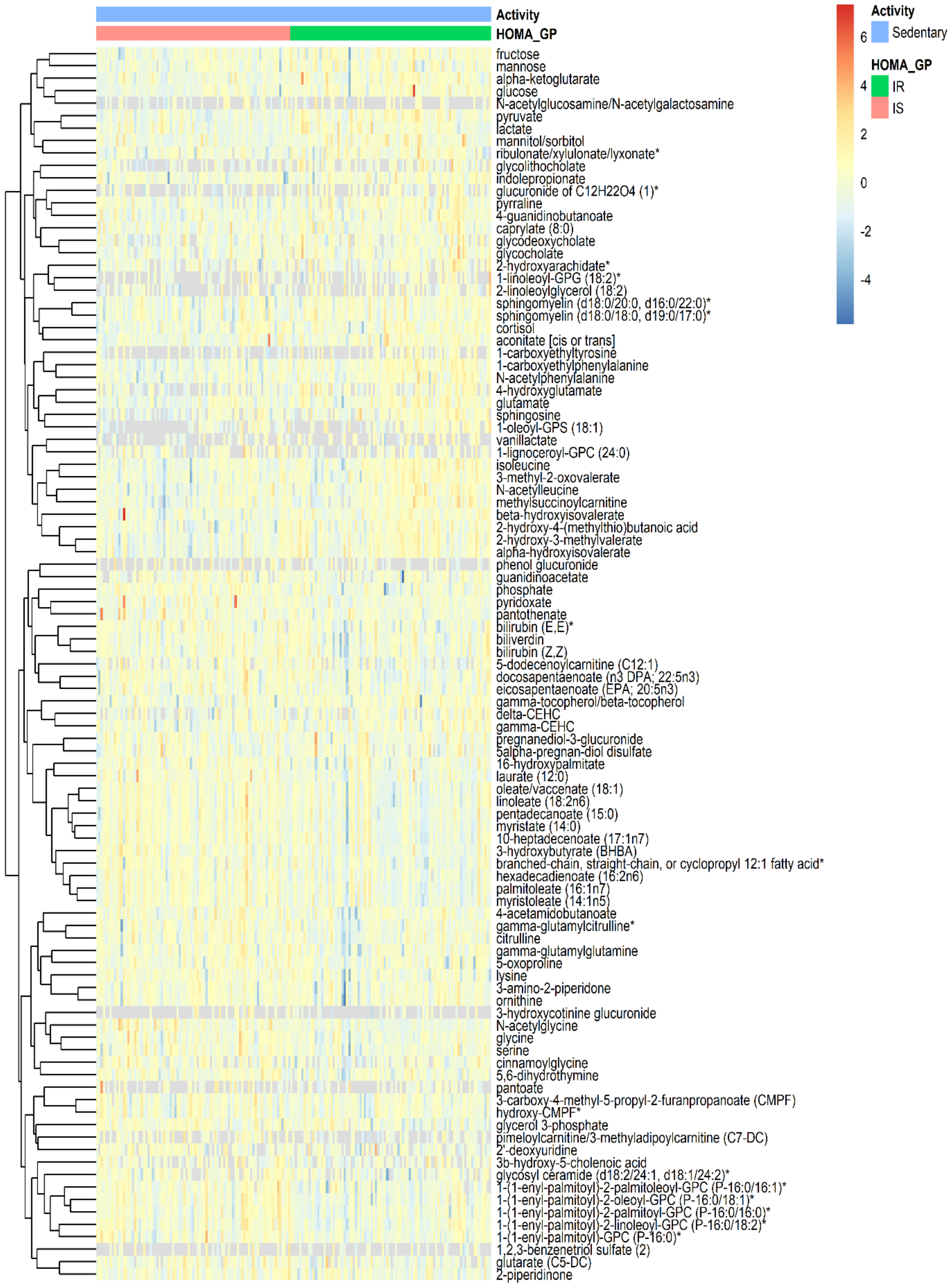

Supplementary figure 3: Heatmap of top 100 metabolites from the univariate analysis that differentiate IS and IR among active individuals.

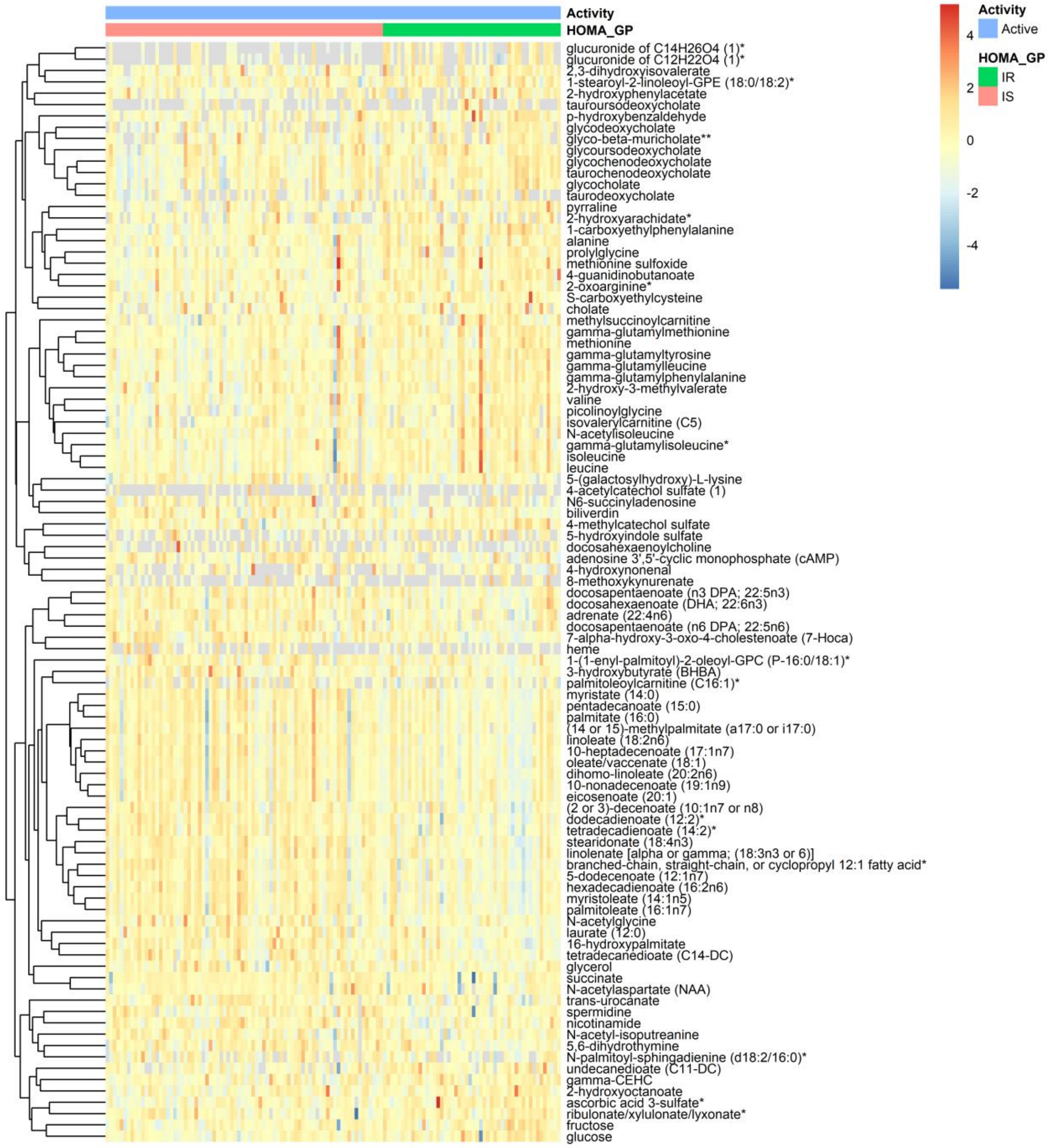

Supplementary figure 4: Volcano plot of metabolites in (a) sedentary (b) active individuals between the insulin sensitive (IS) and the insulin sensitive (IR) groups (Red represents the down-regulated metabolites compared with IR group, green represents the up-regulated metabolites compared with IR group, and gray represents the metabolites with no significant difference between the IS and the IR group.

(a)

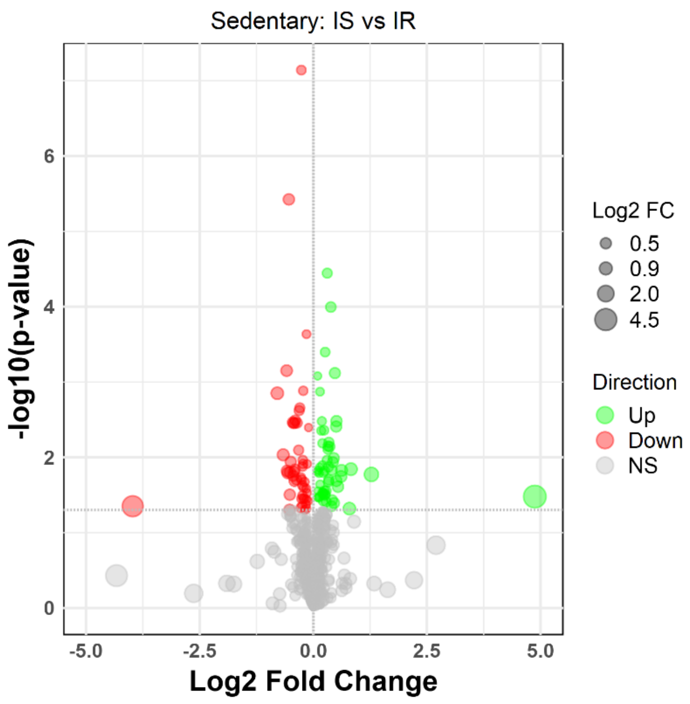

(b)

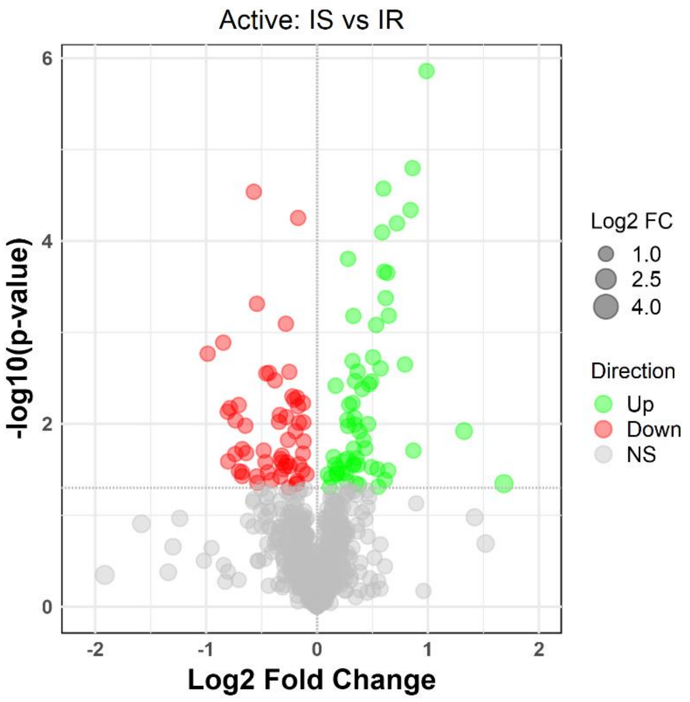

Supplementary figure 5: Scatter plot of metabolites in (a) sedentary (b) active individuals differentiating between the insulin sensitive (IS) and the insulin sensitive (IR) groups from linear regression model after factoring in confounding variables.

(a)

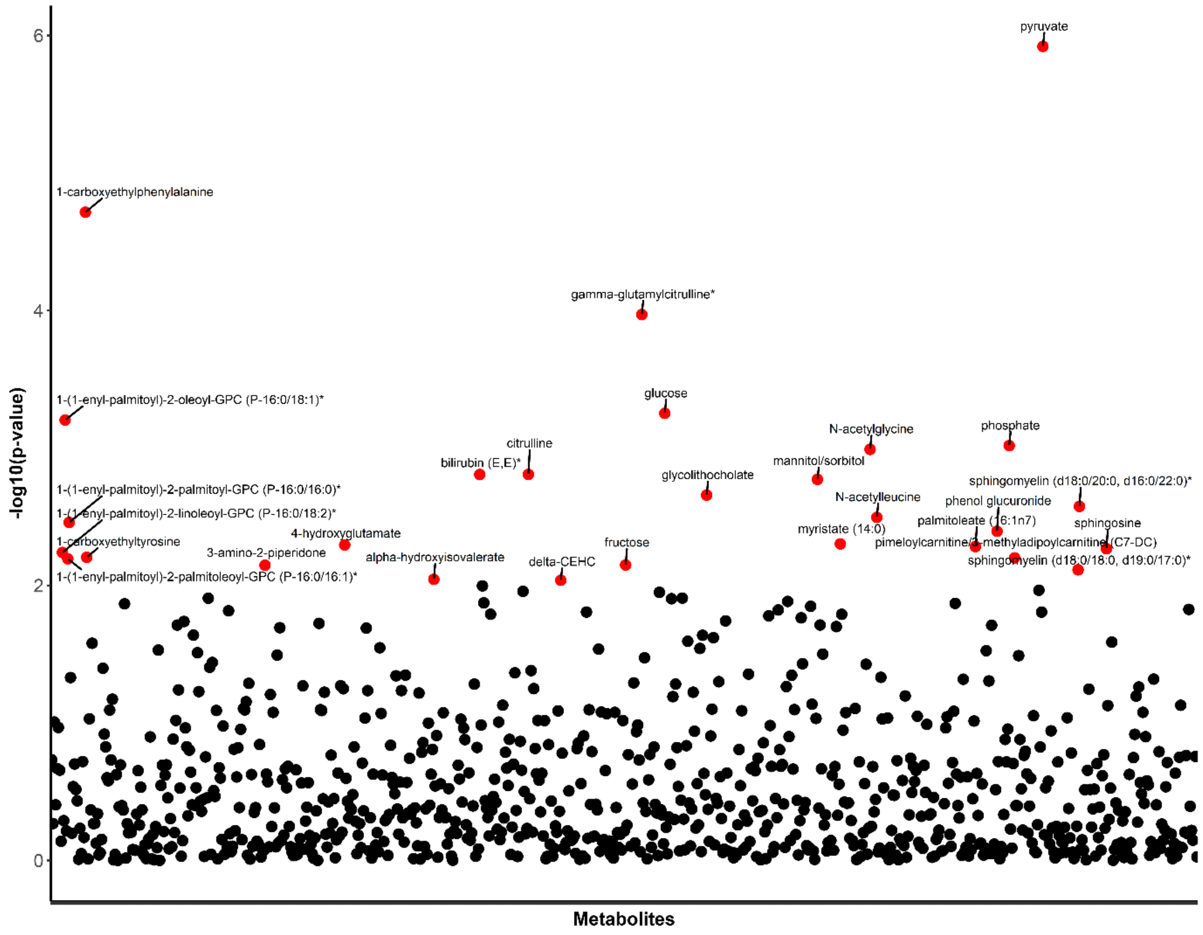

(b)

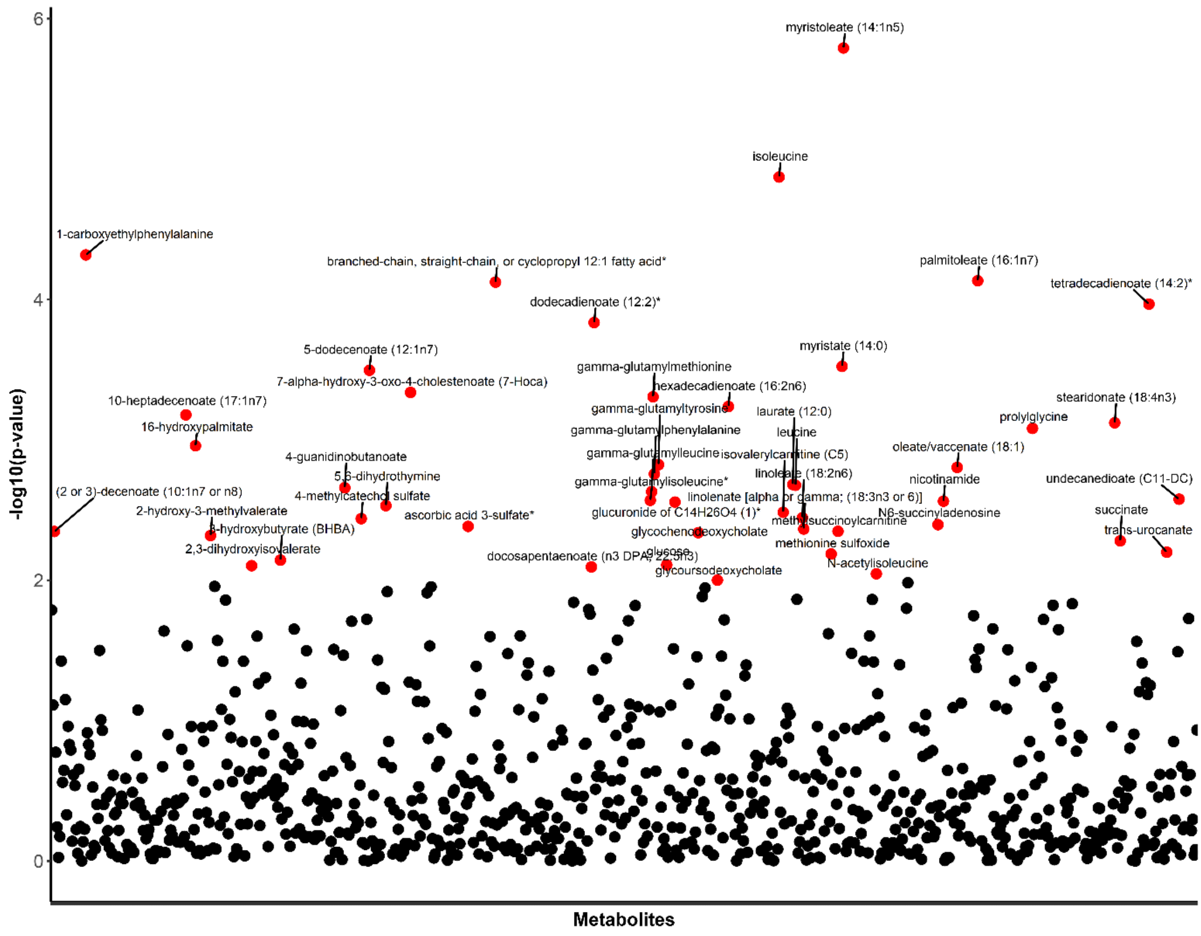
6
4
2
0
-log10(p-value)
Metabolites
myristoleate (14:1n5)
isoleucine
1-carboxyethylphenylalanine
branched-chain, straight-chain, or cyclopropyl 12:1 fatty acid*
palmitoleate (16:1n7)
tetradecadienoate (14:2)*
dodecadienoate (12:2)*
5-dodecenoate (12:1n7)
myristate (14:0)
gamma-glutamylmethionine
7-alpha-hydroxy-3-oxo-4-cholestenoate (7-Hoca)
hexadecadienoate (16:2n6)
10-heptadecenoate (17:1n7)
stearidonate (18:4n3)
gamma-glutamyltyrosine
laurate (12:0)
prolylglycine
16-hydroxypalmitate
gamma-glutamylphenylalanine
leucine
oleate/vaccenate (18:1)
4-guanidinobutanoate
gamma-glutamylleucine
isovalerylcarnitine (C5)
5,6-dihydrothymine
linoleate (18:2n6)
gamma-glutamylisoleucine*
nicotinamide
undecanedioate (C11-DC)
(2 or 3)-decenoate (10:1n7 or n8)
4-methylcatechol sulfate
linolenate [alpha or gamma; (18:3n3 or 6)]
2-hydroxy-3-methylvalerate
glucuronide of C14H26O4 (1)*
ascorbic acid 3-sulfate*
methylsuccinoylcarnitine
N6-succinyladenosine
succinate
3-hydroxybutyrate (BHBA)
glycochenodeoxycholate
trans-urocanate
2,3-dihydroxyisovalerate
methionine sulfoxide
docosapentaenoate (n3 DPA; 22:5n3)
glucose
N-acetylisoleucine
glycoursodeoxycholate

Supplementary table 2: Findings of functional enrichment analysis which was conducted using the Wilcoxon rank sum test on metabolite ranks ranked by p-values (sedentary individuals).

Sub-pathways	p-value	FDR
Long Chain Monounsaturated Fatty Acid	0.001	0.069
Leucine, Isoleucine and Valine Metabolism	0.003	0.141
Hemoglobin and Porphyrin Metabolism	0.004	0.147
Progestin Steroids	0.006	0.150
Urea cycle; Arginine and Proline Metabolism	0.012	0.237
Phenylalanine Metabolism	0.021	0.241
Pantothenate and CoA Metabolism	0.022	0.241
Long Chain Polyunsaturated Fatty Acid (n3 and n6)	0.027	0.249
Guanidino and Acetamido Metabolism	0.029	0.249
Plasmalogen	0.030	0.249

Supplementary table 3: Findings of functional enrichment analysis which was conducted using the Wilcoxon rank sum test on metabolite ranks ranked by p-values (active individuals).

Sub-pathways	p-value	FDR
Long Chain Polyunsaturated Fatty Acid (n3 and n6)	0.000	0.001
Long Chain Monounsaturated Fatty Acid	0.001	0.045
Medium Chain Fatty Acid	0.002	0.066
Leucine, Isoleucine and Valine Metabolism	0.007	0.167
Fatty Acid, Dicarboxylate	0.016	0.311
Primary Bile Acid Metabolism	0.019	0.311
Gamma-glutamyl Amino Acid	0.031	0.387
Pyrimidine Metabolism, Uracil containing	0.031	0.387
Long Chain Saturated Fatty Acid	0.040	0.414
Creatine Metabolism	0.041	0.414
Ketone Bodies	0.050	0.459
